# Ultrasound-Guided Interscalene Brachial Plexus Block for Pathological Humerus Fracture due to Multiple Myeloma with Systemic Manifestation: Useful Option for Management in Low-Income Countries

**DOI:** 10.1155/2020/9892580

**Published:** 2020-10-17

**Authors:** Pawan Kumar Hamal, Bibena Lamichhane, Nabin Pokhrel, Janith Singh, Rupesh Kumar Yadav

**Affiliations:** National Academy of Medical Science, National Trauma Center, Kathmandu, Nepal

## Abstract

Anesthetic management of pathological fracture due to multiple myeloma with systemic manifestation poses a perioperative challenge especially in low-resource setups like Nepal. Regional anesthesia using ultrasound-guided block can improve the accuracy, reduce complications, and improve overall perioperative management of pathological fractures due to malignancy with systemic spread in resource-deprived setups. We present a case of a 53-year-old lady with pathological fracture of left humerus shaft, a diagnosed case of multiple myeloma with compression fracture of multiple lumbar spine with chest wall metastasis with resolving acute kidney injury with chest infections. Ultrasound-guided interscalene brachial plexus block with sedation was done for open reduction internal fixation of humerus shaft fracture taking in consideration the overall high perioperative risk of patient. Intraoperative hemodynamic was uneventful, with no neurological sequelae and good recovery status perioperatively. Ultrasound-guided interscalene brachial plexus block if done cautiously can be a very useful alternative technique for better perioperative outcome in patients with malignancy with systemic spread in areas where expertise is scarce and resource is limited.

## 1. Introduction

In low-income countries like Nepal, where the anesthesia specialty is in growing stage, managing pathological fractures due to multiple myeloma with systemic spread poses a perioperative challenge [1]. Regional anesthesia using ultrasound-guided block can improve the accuracy, reduce complications, and improve overall perioperative management of pathological fractures due to malignancy with systemic spread [2–4]. We present a case of a 53-year-old lady with pathological fracture of left humerus shaft, a diagnosed case of multiple myeloma with compression fracture of multiple lumbar spine with chest wall metastasis with resolving acute kidney injury with chest infections. Ultrasound-guided interscalene brachial plexus block (IBPB) with sedation was done successfully for open reduction internal fixation of humerus shaft fracture taking in consideration the overall high perioperative risk.

## 2. Case Presentation

A 53-year-old lady from Kathmandu presented with complaints of pain and swelling over the left arm following fall on the ground. She also complained of on and off productive mucoid cough since last six months. There is no history of chronic obstructive pulmonary disease, pulmonary tuberculosis, hypertension, diabetes mellitus, or any other illness. She also had swelling over the left chest which was diagnosed based on tissue biopsy as small round-cell tumor of the chest wall. She had also undergone chemotherapy with dexamethasone one month prior to injury. She had a history of on and off backache in the thoracolumbar area, nonradiating, intermittent, relieved by analgesics, and aggravated by mild exercise but with no history of trauma. She was also a known case of somatoform disorder under clonazepam.

**Figure 1:**
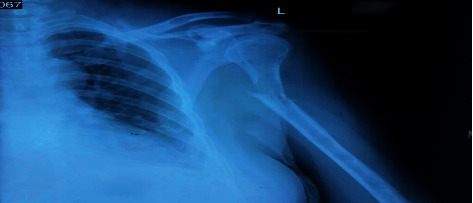
Humerus fracture with lytic lesion.

**Figure 2:**
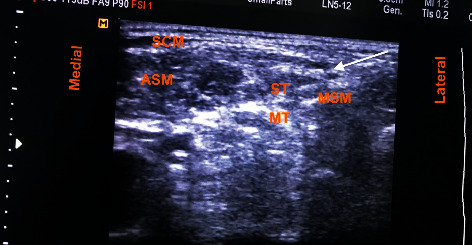
Preliminary scan showing brachial plexus in the interscalene groove. Arrow indicates needle direction. Abbreviations: ASM, anterior scalene muscle; LA, local anesthesia; MSM, middle scalene muscle; MT, middle trunk; ST, superior trunk; SCM, sternocleidomastoid muscle.

**Figure 3:**
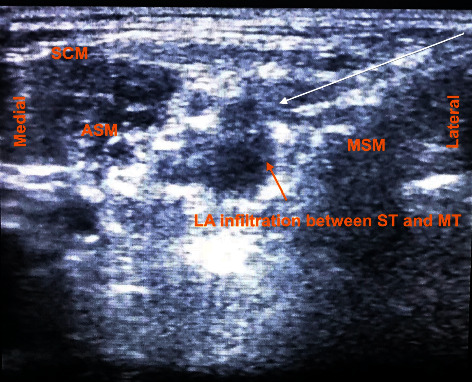
Local anesthesia spread around the brachial plexus trunk with needle. Arrow indicates needle direction. Abbreviation: ASM, anterior scalene muscle; LA, local anesthesia; MSM, middle scalene muscle; MT, middle trunk; ST, superior trunk; SCM, sternocleidomastoid muscle.

On examination, her vitals were stable, and other systemic examination was within normal limit. Airway examination showed short neck and Mallampati of grade III. Local examination revealed swelling and tenderness over the left midarm. Her initial laboratory investigations showed increased total count with neutrophilic predominance and thrombophilia. Serum calcium was normal, but serum phosphorous was low (1.5 mg/dl). Her serum creatinine was raised to 7.5 mg/dl with blood urea of 106 mg/dl. Urinary Bence Jones protein was positive. Immunochemistry revealed high beta 2 microglobulin (13616 ng/ml), high alpha 1 and 2 globulin (0.56 g/dl and 1.08 g/dl, respectively), and low serum albumin (3.09 gm/dl). Noncontrast computed tomography showed multiple lytic lesions in the body of thoracic vertebra with a collapse of D8 vertebra. She was hence diagnosed as multiple myeloma, admitted to the hospital, and optimized. On preanesthesia evaluation, the total count was high (16,400/mm^3^); hemoglobin, 8.3 gm%; thrombophilia, 5,98,000/mm^3^; and prothrombin time 12.4 seconds with international normalized ratio of 1.00. Her acute kidney injury (blood urea 50 mg/dl and creatinine 1.6 mg/dl) was resolving few days after admission. Serum sodium was 137 meq/L, and potassium was low (2.4 meq/L). SPO_2_ was 86–90% with PO_2_ of 66.2 mm Hg in room air. Liver functions were normal except for high lactate dehydrogenase (427 IU/L). Echocardiography revealed normal cardiac function with no underlying structural abnormality and ejection fraction of 60%. X-ray revealed fracture shaft of humerus with multiple lytic lesions (Figure 1). Preliminary diagnosis of American Society of Anesthesiologist (ASA) Physical Status Grade III with left pathological humerus fracture with multiple myeloma with chest wall metastasis with resolving acute kidney injury with somatoform disorder with probable difficult airway was made. She was then planned for open reduction and internal fixation with PHILOS plating. Challenges of increased risk for perioperative bleeding, chest infections, postoperative mechanical ventilation, deep vein thrombosis, and local anesthesia toxicity were anticipated. Therefore, we planned her under regional anesthesia with IBPB under ultrasound guidance with sedation. Even with IBPB, the risk of neurological sequelae particularly phrenic nerve blockade leading to respiratory compromise in the patient was possible. Possibility of requirement of general anesthesia (GA) and postoperative mechanical ventilation was also discussed. Written informed consent with the patient party was taken. On the day of surgery, in the operation theatre, a monitor was attached, and baseline vitals were taken. Ceftriaxone 1 g injection was given intravenously as prophylactic antibiotic. Preliminary scan was done to visualize brachial plexus in the interscalene groove (Figure 2) with ultrasound (Samsung My SONO U6) with linear probe. Location of the phrenic nerve was scanned between the anterior scalene and sternocleidomastoid muscle. Using the in-plane technique and taking care of asepsis, ultrasound compatible needle (PAJUNK, SONOPLEX STIM, 20G × 150 mm) was used to infiltrate the three trunks, mostly superior and middle trunk and lower trunk the least (Figure 3), with total 20 ml local anesthetic (Two 10 ml syringe each containing 5 ml of 1% lignocaine with adrenaline (1 : 100000) and 5 ml of 0.25% bupivacaine plain after dilution). At the time of injection and spread of local anesthetics, caution was taken to visualize the tip of the needle below the superior trunk in the interscalene groove and away from the plane of anterior scalene and sternocleidomastoid to avoid phrenic nerve blockade (Figure 3). At the same time, extra care was taken to use as minimum injection pressure as possible while injecting 5 ml aliquots of local anesthesia. Sensory block was ensured with the pinprick method in the related dermatome. Motor block for radial, ulnar, and median was also present, with minimum handgrip. Clinically, respiratory movement was symmetrical bilaterally and adequate to maintain the oxygen saturation. However we did not measure the diaphragmatic excursion on the blockade side or did any pre- and postspirometry tests to notice the changes. Dyspnea, hoarseness of voice, hiccups, or features of Horner's syndrome were not noted. The patient was sedated using propofol injection at a rate of 50 mcg/kg/min via an infusion pump, and supplemental oxygen was given via facemask at 5 L/min. Fentanyl 50 mcg injection was also given intravenously prior to block to ensure proper pain management. Her vitals remained stable throughout the intraoperative period of 3 hours, and her urine output was maintained 1 ml/h (total 150 ml). Postoperative pain catheter was not placed due to lack of equipment, inadequate training, untrained nursing staff, and risk of high dose of local anesthetics aggravating kidney injury in the postoperative period due to prolonged infusion. In the postoperative ward, pain was managed using multimodal approach with paracetamol 1 g injection every 6 hours and fentanyl infusion at 20 mcg/h. Supplemental oxygen was continued for another 6 h postoperatively at the rate of 2 L/min via nasal prongs, and incentive spirometry was advised. Her Numeric Rating Pain (NRS) scale remained less than 4 out of total 10 mm throughout the first operative day. Complaints of dyspnea, hoarseness, hiccups, and signs of Horner's Syndrome were also not present during the same period. On the second postoperative day, paracetamol was continued with fentanyl as per required basis. Her NRS rating was 2 to 3 and was ambulating comfortably. She had early enteral feeding, mobilization with good compliance on incentive spirometry on the first postoperative day with no issues of postoperative nausea and vomiting. She was discharged on the 5^th^ postoperative day with no issues of wound infections, and her renal function stayed with the normal limits.

## 3. Discussion

In the last one decade, there is a rising trend of diagnosed multiple myeloma as the population are aging in low-income countries like Nepal [1]. Most of them present with pathological fractures with multiple systemic complications with poor outcome due to lack of proper access to care [5]. Almost 80% of patients at diagnosis have osteolytic lesions that increase the risk of pathological fractures [6], reduce the quality of life [7], and increase the treatment costs [8]. Approximately 60% of myeloma patients will develop a fracture during the disease course [9]. This further puts a greater challenge to a budding group of anesthesiologists in low-income countries with limited expertise in ultrasound-guided regional anesthesia to provide safer option and good perioperative care and pain management.

The pathogenesis of disease defines multiple perioperative considerations such as pathological fractures, renal failure, bleeding diathesis, increased susceptibility to infections, hypercalcemia, anemia, deep venous thrombosis, hyperviscosity syndrome, embolism, and delayed wound healing [3, 10, 11]. If the patient is under chemotherapeutic regimen, the risk increases and make [3] the management even more difficult. The overall mortality risk in our patients according to ASA physical status grade for elective surgery was 1.4% [12]. International staging system for multiple myeloma calculates the medial overall survival for this patient as 44 months [2]. On the other hand, the patient developed somatoform disorder which was although managed with clonazepam tablet for few days, making it difficult to perform the procedure when patient was awake. The patient had short neck, making the placement of the probe and visualization of the brachial plexus difficult. Local anesthesia toxicity has been reported even at usual safe dose limit in patients with raised alpha glycoprotein level and in multiple myeloma patients undergoing ultrasound interscalene block [13]. Our patient also had the risk of increased local anesthesia toxicity. However, the patient did not manifest it which can partly be explained by use of local anesthesia limited to the intraoperative period. Local anesthetic drugs are mostly metabolized in liver and can be a good choice for patient with deranged renal functions. Bupivacaine used for 48 hours even at concentration of 0.5% has been shown to be less injurious even to ischemic kidney tubules in animal studies [14]. Patient-controlled analgesia infusion in IBPB using 0.15% bupivacaine or ropivacaine provides adequate pain relief, similar side effect profile, and high patient satisfaction after shoulder surgery which can be a useful addition in these contexts too [15].

To avoid phrenic nerve blockade, use of low volume, low concentration, and local anesthetics have been recommended. IBPB was performed with isolating superior trunk and limiting potential spread to the phrenic nerve, and the recurrent laryngeal nerve has demonstrated less subjective dyspnea, hand immobility, and hoarseness with high patient satisfaction scores postoperatively [16]. We did take cautions of isolating the phrenic nerve, injecting below the superior trunk, and lowering the concentration of local anesthetics to a safety level compatible to renal function and also avoiding blockade of phrenic nerve demonstrated clinically. Although lowering the volume to as low as 10 ml has been suggested as useful strategy for similar analgesic efficacy, they were done under IBPB with GA, which can be a poor choice in our case given various issues related to GA and possibility of longer surgical duration. The use of GA technique on the other hand would have increased the risk of venous thrombosis, hemodynamic instability, postoperative renal failure, and delayed wound healing, making the management even more difficult. Studies done with 20 ml volume compared to conventional 40 ml of local anesthetics for IBPB demonstrated similar hand strength grip, lesser decrease in negative inspiratory pressure postoperatively, and lesser incidences of hoarseness with similar satisfaction and analgesia [17]. With sole use of nerve blockade technique, a volume of 16–20 ml of local anesthetics had shown to have lower incidences of postoperative neurological outcome [18]. Limiting volume of local anesthetics is likely to spare suprascapular nerve which is likely to arise from the superior trunk and can be an important innervation in shoulder surgery [19]. Doubling the volume to 60 ml and halving the local anesthetic concentration to 0.25% bupivacaine with adrenaline and 1% lignocaine has been shown to have no incidence of phrenic nerve using the nerve stimulator technique, surprisingly with almost equivalent analgesic efficacy, patient comfort, and satisfaction [20]. Care was also taken in our case not to infiltrate the lower trunk too much, preserve hand motility, and improve patient satisfaction score [16]. Additionally, ultrasound-guided IBPB improves the onset of drug action and accuracy and reduces risk of hematoma and pneumothorax compared to the conventional regional anesthesia technique [4]. It also reduced the opioids requirement, chances of delayed recovery produced by use of sedating agents, muscle relaxants, and induction agents. It has been a modality used in well-equipped center for the management of pathological fracture due to multiple myeloma [3, 21]. Properly used ultrasound-guided IBPB taking cautions not to block the phrenic and the recurrent laryngeal nerve provides the better options in setting with limited expertise in low-income countries for the management of multiple myeloma with systemic manifestation and improves the overall perioperative outcome.

## 4. Conclusions

Ultrasound-guided interscalene brachial plexus block provided better alternative to general anesthesia for patients with pathological humerus fracture due to multiple myeloma with systemic manifestation and chest infections in low-income country setups with limited expertise. Care should be taken while choosing appropriate drug, volume, concentration, and technique using ultrasound guidance to avoid phrenic nerve palsy in patients with multiple comorbidities. It helps improve overall perioperative outcome in high-risk surgery.

## Data Availability

Data can be shared upon reasonable request with consent from the patient party.
